# Telemetric lumbar cerebrospinal fluid pressure monitoring for spaceflight: a novel approach to spaceflight-associated neuro-ocular syndrome research

**DOI:** 10.1038/s41526-026-00586-0

**Published:** 2026-05-07

**Authors:** Anders Eklund, Tomas Bäcklund, Michael A. Williams, Jan Malm

**Affiliations:** 1 Department of Diagnostics and Intervention, Biomedical Engineering and Radiation Physics, Umeå University, Umeå, Sweden; 2 University of Washington School of Medicine, Seattle, WA USA; 3 Department of Clinical Science, Neurosciences, Umeå University, Umeå, Sweden

**Keywords:** Engineering, Health care, Medical research, Neurology, Neuroscience

## Abstract

Spaceflight-associated neuro-ocular syndrome (SANS) poses a significant risk for astronauts, being linked to intracranial pressure (ICP) changes in microgravity. Investigating ICP in space has been challenging. We evaluated a telemetric lumbar ICP monitoring system that shows promise for investigating microgravity-induced intracranial pressure changes in astronauts. Despite limitations in pulse amplitude analysis and pressure underestimation, the system’s long-term stability and overall performance support its recommendation for SANS research in space.

## Introduction

With the emergence of Spaceflight Associated Neuro-ocular Syndrome (SANS), understanding the dynamics of intracranial pressure (ICP) in astronauts has become crucial^1,2^. SANS presents with symptoms such as disc edema, choroidal folds, and globe flattening, similar to idiopathic intracranial hypertension (IIH) on Earth. However, the absence of headaches, typically associated with IIH, suggests that while the etiology of SANS may be similar, it is not identical^3^.

Measuring ICP in microgravity is key to understanding SANS. ICP is influenced by pressure in the cerebral venous sinuses, internal jugular veins, and central venous pressure (CVP)^4^. On Earth, the venous system is sensitive to gravity, and dural sinus venous pressure varies with posture, lower when upright^5^, resulting in slightly negative ICP^6^ compared to around 11 mmHg in the supine^7^. In microgravity, these hydrostatic effects disappear, rendering constant venous pressure regardless of position^8,9^. Consequently, ICP in space is expected to be higher than upright Earth values but comparable to supine ICP in 1G^8^. Prolonged missions induce optic nerve elongation, a potential consequence of elevated 24-h ICP averages^10^. This elongation may exert mechanical pressure on the eye, leading to globe flattening, choroidal folds, and possibly edema. To investigate SANS, ICP must be measured in microgravity.

Although ICP dynamics in space remain poorly understood, astronauts returning with SANS symptoms and show elevated ICP more often than expected from normal adult ICP distribution^11–13^. Short-term ICP recordings during parabolic flight suggest modest ICP reduction in microgravity^9^, yet levels remain higher than those observed upright on Earth, highlighting the need for ICP monitoring in space.

Additional fluid dynamics parameters that change with mean ICP, posture, and elevated carbon dioxide include craniospinal compliance and cerebral blood flow pulsatility^14^. Cephalad fluid shifts in microgravity may reduce compliance^15^, increasing ICP pulse amplitude and altering CSF flow patterns, potentially creating local intracranial pressure gradients relevant to SANS. Investigating changes in craniospinal compliance by monitoring ICP pulse amplitude and its relationship to mean ICP is therefore important.

Investigations into ICP among astronauts have faced challenges. Non-invasive methods lack accuracy^16–18^. Lumbar puncture, a standard method for ICP assessment, is not feasible in space^19^. Implantable ICP sensors offer promise but currently require ventricular catheters and neurosurgery. A less invasive option would be inserting a lumbar catheter and attaching it to a telemetric ICP monitoring device.

Since sample sizes will be small in astronaut studies, it is essential to understand the capabilities of a telemetric system. Given the novelty and complexity of using a lumbar catheter-based telemetric system in healthy astronauts, rigorous bench testing ensures reliability and safety under controlled conditions and informs future development of implantable monitoring technologies for spaceflight.

In this bench test study, we assessed the accuracy and hydrodynamic characteristics of a complete telemetric pressure measurement system including a lumbar catheter set from Medtronic, connected to a Miethke M.scio pressure transducer and a telemetric transmission data system for recording ICP (P_M_). We specifically investigated its performance in detecting mean ICP and pulsatile variations in ICP against a reference pressure (P_Ref_).

**Fig. 1:**
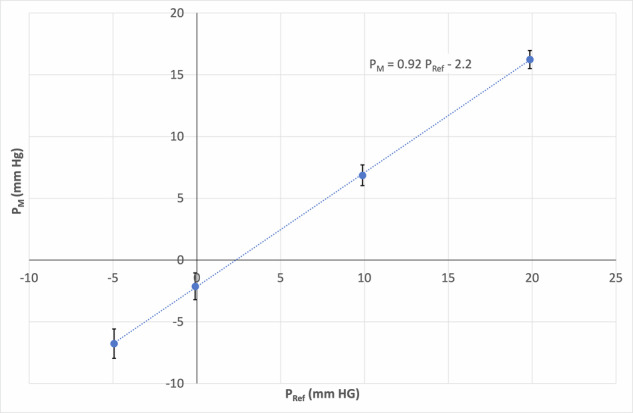
Static pressure as measured with the P_Ref_ and the P_M_. Although we found a general underestimation, there is a good accordance against the reference method, specifically with respect to detecting a within subject change in pressure.

We present data from six M.scio sensors investigated for frequency response curves, including the effect of different catheter lengths. We also evaluated accuracy in measuring mean pressure and capability to detect typical ICP pressure waves. The assessment of static mean pressure on the 6 sensors showed acceptable agreement between P_M_ and P_Ref,_ with a mean difference (P_M_-P_Ref_) −2.62 ± 1.21 mmHg, *N* = 96 (*p* < 0.001, paired *t*-test). The difference was significant and P_M_ underestimated P_Ref_ at all four static pressures: −5 mmHg (−1.83 ± 1.18 mmHg), 0 mmHg (-2.03 ± 1.08 mmHg, *N* = 24), 10 mmHg (-3.02 ± 0.83 mmHg, *N* = 24) and 20 mmHg (−3.63 ± 0.72 mmHg, *N* = 24)(Fig. 1). The pattern was similar for all six sensors with a 90–95% change in P_M_ compared to P_Ref_. Variability (noise) of P_M_ when measuring on a constant hydrostatic column was similar on all levels, with a standard deviation of ~0.38 mmHg. The corresponding variability for P_Ref_ was 0.01 mmHg, showing that the actual pressure was very stable. Examples of this difference in noise level between P_M_ and P_Ref_ can be seen on the static levels in both Figs. 6 and 7 in the method section.

**Fig. 2:**
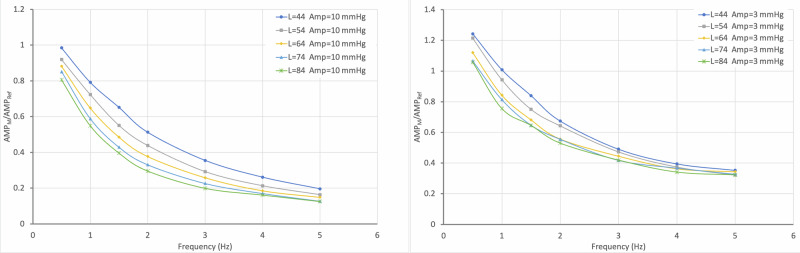
Frequency response curve for the M.Scio with a Medtronic lumbar catheter of length 44–84 cm with amplitudes of 10 mmHg (left) and 3 mmHg (right)*.* Note that for 1 Hz, which is close to the cardiac frequency, ~80% of the pulse amplitude was recorded with the M.Scio for the 44 cm catheter. For frequencies above 2 Hz the amplitudes are heavily dampened. The tendency of a higher AMP_M_ amplitude transfer with 3 mmHg ref amplitude can most likely be explained by the noise noted in the P_M_ signal that will cause an addition to both max and min points of each wave period and thereby an incorrectly elevated amplitude.

We investigated the frequency response of the telemetric system with different catheter lengths using sinusoidal pressures with peak to trough amplitudes (AMP) of 3 or 10 mmHg in a pressure chamber and assessing AMP_M_/AMP_Ref_. Figure 2 presents the frequency response for the Miethke M.scio with a Medtronic lumbar catheter of 44, 54, 64, 74 and 84 cm lengths. Analyzing the 10 mmHg AMP (Fig. 2A), dampening was pronounced, with amplitude reduced to 80% at 1 Hz for the 44 cm catheter and to 55% for the 84 cm catheter. For 5 Hz, amplitude was reduced to less than 20% for all catheter lengths. For the 3 mmHg AMP (Fig. 2B), the M.scio noise level described above always added significantly to the true AMP signal, generating an overestimation of ~20%. This add-on was seen for all frequencies including the 0.5 Hz frequency where the noise generates an overestimation of the amplitude (Fig. 2B).

In one measurement series with a 44 cm catheter that was excluded from the study, with AMP = 5 mmHg we saw major increased dampening with less than 50% of amplitude remaining at 1 Hz. We suspected a tiny air bubble in the dome of the M.scio and therefore flushed the system and repeated the measurement series. We then had a much better amplitude response for all frequencies. This informs us that at the time of implantation of the telemetric ICP sensor in human subjects, great care must be taken to remove all bubbles from the sensor before the surgical wound is closed. Otherwise, damping of the ICP pulse pressure could lead to underestimation of the true ICP pulse pressure.

**Fig. 3:**
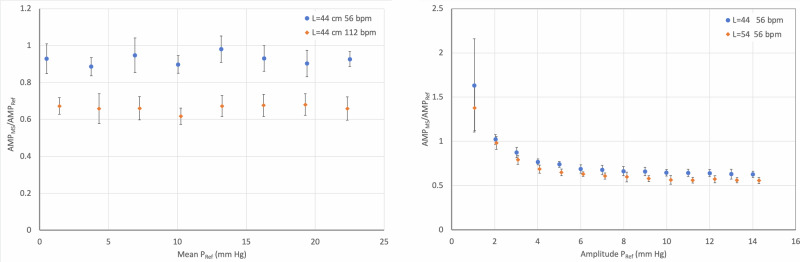
Amplitude dampening with respect to mean reference pressure and reference amplitude. Show the damping of ICP with different heart rates (56 resp. 112 bpm) at different mean pressures with a pulse amplitude of 3 mmHg (left). Show the damping of the ICP with different catheter length, 44 resp. 54 cm at different peak to peak pulse amplitudes (1–14 mmHg) with a mean pressure of 11 mmHg (right).

The assessment with simulated ICP signal and two different heart rates confirmed increased dampening with higher heart rate (Fig. 3A). Experiments with different amplitudes with HR = 56 and 112 bpm showed that amplitude did not affect the amplitude response, except for the expected effect of the M.scio noise which becomes relevant for amplitudes below 4 mmHg (Fig. 3B).

**Fig. 4:**
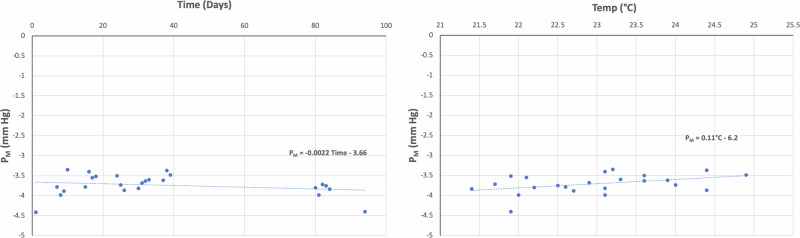
Sensor drift with respect to time and temperature. Mean long-term drift for the six sensors for 94 days (left). Mean temperature drift for the six sensors at variable room temperature (right).

In the 90-day long-term test we saw no systematic drift of P_M_ readings (*r* = 0.24, *p* = 0.25) (Fig. 4A). Furthermore, the assessment with respect to variation in ambient room temperature showed good stability but with a small significant increase of measured pressure with increased ambient temperature (*r* = 0.42, *p* = 0.05) (Fig. 4B). The M.scio TRU offered readings of the ambient pressure which was compared to the atmospheric pressure recorded at the local weather station, we found a good agreement *r* = 0.99, but the pressure response in the TRU was 97% of the actual change. The assessed *P*_*M*_ in long-term test with respect to atmospheric pressure showed no association (*r* = 0.12, *p* = 0.58).

In this study, we have investigated a telemetric pressure measurement method intended for lumbar catheter access to the lumbar cerebrospinal fluid (CSF) space of an astronaut before, during, and after spaceflight. Specifically, our aim was to evaluate its accuracy and dynamic properties prior to implantation so that its response characteristics were understood and could be corrected for in relation to the reference pressure, thus providing a more accurate measurement of ICP during spaceflight. This is a first necessary step towards providing insights into the mechanisms underlying SANS and contributing to the development of more effective preventative measures and treatment strategies for astronauts during extended space missions.

The assessment of the telemetric ICP system’s performance is crucial for designing in vivo experiments and interpreting their results. In our analysis, utilizing M.scio sensor and Medtronic lumbar catheter (44410), we made several significant findings. While the telemetric system demonstrated reproducible mean pressure assessment, it consistently underestimated pressure and detected only 90–95% of the change in reference pressure. Since we primarily will be interested in within-patient changes, this is acceptable and can be adjusted for. To ensure accurate ICP measurements, we recommend conducting bias estimation before and after spaceflight using an external pressure measurement connected with a needle into the M.scio sensor’s dome.

Regarding dynamic behavior, we observed significant dampening of pulse pressure from the catheter, particularly evident in longer catheters. This dampening effect must be considered when analyzing pulse amplitude changes, especially concerning variations in heart rate. Due to the significant noise level in the M.scio measurements, which adds a non-physiological component to amplitude estimation, a low-pass filtering of data with a cutoff frequency of ~3–5 Hz is essential for detecting reliable amplitudes. This is particularly important for subjects with amplitudes in the range of 3 mmHg and lower, which can be expected in healthy astronauts.

It is important to note that the M.Scio sensor coupled with the Medtronic 44410 catheter may experience substantial pressure dampening for frequencies beyond those corresponding to the cardiac cycle. However, slower ICP variations, such as B-waves and respiratory waves, remain analyzable if their amplitudes exceed 2 mmHg. Attention to detail during sensor implantation, including the removal of air bubbles, is paramount to ensuring accurate pressure measurements.

Tests of stability over time demonstrated that the M.scio sensors exhibited no systematic drift, essential for tracking changes in astronauts, since those enduring extended microgravity exposure are most likely to develop SANS^3^. Additionally, while a slight correlation between measured pressure and ambient temperature was observed, its clinical relevance is likely insignificant given the stable temperature environment within patients.

In conclusion, the telemetric pressure recording system, comprising the M.Scio sensor and the Medtronic 44410 lumbar catheter, shows promise for investigating microgravity-induced intracranial pressure changes. From the characteristics determined in this study limitation in pulse amplitude analysis and the system’s overall performance are known and can be accounted for, and together with good long-term stability this support its recommendation for studies involving astronauts during space missions.

## Methods

**Fig. 5:**
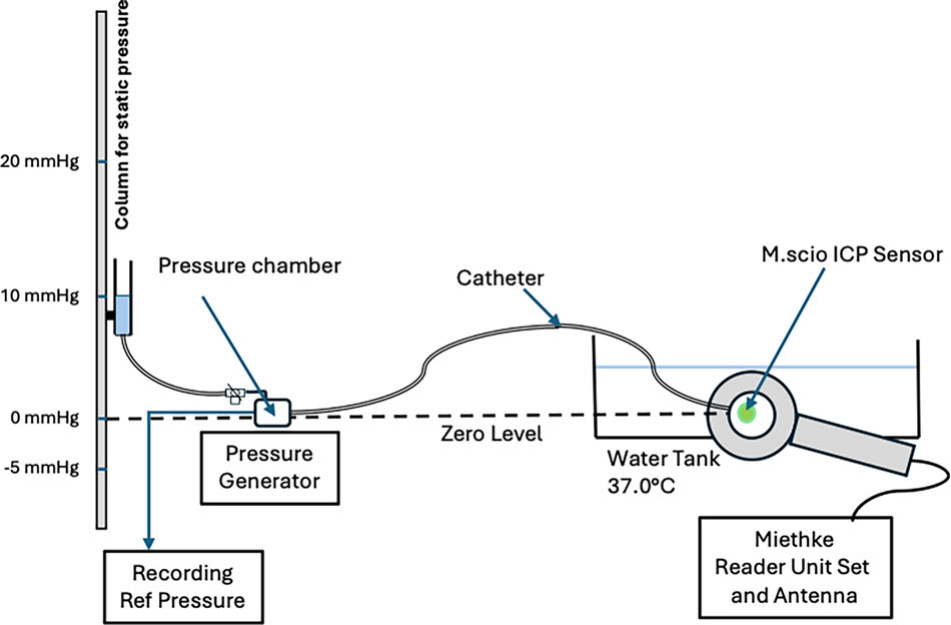
Schematic drawing of the experimental set-up.

We investigated the pressure measurement characteristics of six new M.Scio telemetric sensor (Miethke GMBH, Germany) and the corresponding Telemetric Reader Unit (TRU). Since we were aiming for a lumbar application, the M.Scio was evaluated together with six Medtronic 44410 lumbar catheters (Medtronic, Minneapolis, MN, USA). To connect the narrow diameter lumbar catheter to the standard hub on the M.Scio a step-up connector (Medtronic, Product Number 22063) and a short catheter segment were used. The telemetric pressure recorded from the M.Scio system is referred to as P_M_. The static and dynamic characteristics of the pressure measurement system were evaluated in a bench test (Fig. 5). Pressure waves were generated in a fluid filled pressure chamber by a pressure wave generator (Model 601A Blood pressure system calibrator, Biotech Instruments, Inc., Burlington, VT, USA). Pressure variation was created with a signal protocol designed in LabView (National Instruments Corporation, Austin, TX, US), generated using a data acquisition card NI-DAQ NI6011E (National Instrument), and fed to the pressure wave generator. The TRU was set at the “Fast” measurement mode with a verified sample rate of 46 Hz for acquisition of P_M_.

Reference pressure (P_Ref_) was assessed with a standard pressure transducer (PMSET 1TNF-R, BD Critical Care Systems Pte Ltd, Singapore) attached to an in house developed pressure amplifier including a low pass filter with cutoff frequency of 20 Hz. The reference system was connected directly to the fluid chamber. Reference pressure was sampled at 100 Hz using a data acquisition card NI USB 6216 (National Instruments) and software developed in LabView. The whole system was filled with purified water. To fill the system, all air was removed by carefully flushing the system. To clear the M.Scio sensor of air bubbles, the dome on the sensor was gently tapped while flushing until no air was seen.

We used three different protocols to investigate 3 aspects of the telemetric sensor/catheter system: 1) mean pressure accuracy and the influence of mean pressure on amplitude response; 2) the influence of catheter length on amplitude response; and 3) the stability of the system over three months.

**Fig. 6:**
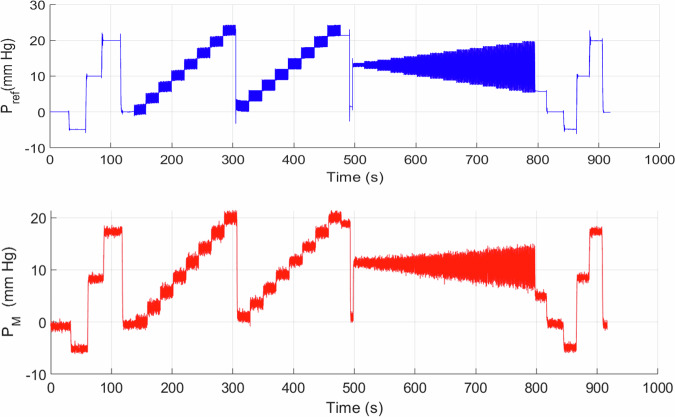
Top row shows P_Ref_ and bottom row P_M_. The series starts and ends with four hydrostatic pressure levels and the response with increased mean pressure from 0 to 21 mmHg in 3 mmHg steps at 56 and 112 BPM, respectively. Thereafter, we investigated the response to different amplitudes by varying the amplitude from 1 to 14 mmHg in step of 1 at 56 BPM and a mean pressure of about 11 mmHg.

In the first protocol we investigated the variability between sensors. Six M.Scio sensors were tested for both static and dynamic characteristics. The sensors were at all times in a water bath controlled at 37.0 °C with a Steba SV50 (Steba Elektrogeräte GmBH, Germany) circulator. The protocol, presented in Fig. 6, was started and ended with a test of static mean pressure test, utilizing a hydrostatic column to adjust pressure sequentially from 0 to −5, 10, and 20 mmHg (Fig. 5). Pulsatile pressure assessment was tested with an ICP-waveform reproduced from a healthy volunteer recording^8^. With an input amplitude (always defined as peak to trough) of 3 mmHg, we assessed the influence of mean pressure by varying mean pressures from 0 to 21 mmHg in 3 mmHg increments, at both normal (56 bpm) and double heart rate (112 bpm). In addition, we investigated the response to different amplitudes by varying the amplitude from 1 to 14 mmHg in steps of 1 mmHg, having a constant mean pressure of 11 mmHg and heart rate of 56 bpm. The whole protocol was carried out for two catheters, one 44 cm and one 56 cm. These catheter lengths were based on estimates with the M.scio transducer implanted in the mid-axillary line, at least 10 cm of catheter within the lumbar CSF space, and enough catheter length to prevent pulling or disconnection of the catheter from the M.Scio hub. Figure 6 presents the protocol by showing typical measurements with the reference system and the M.Scio system from one acquired measurement series.

**Fig. 7:**
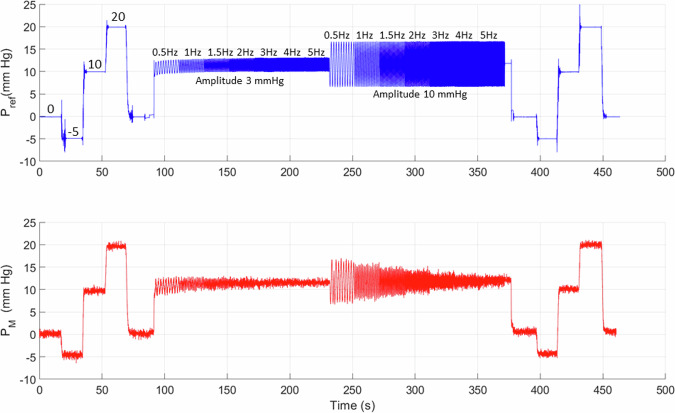
Top row shows P_Ref_ and bottom row shows P_M_. The series starts and ends with the four hydrostatic pressure levels (0, −5, 10, 20 mmHg) and then intervals with sinusoidal pressure variations from 0.5 Hz to 5 Hz. In this specific series there was mean pressure of about 11 mmHg and a peak-to-peak amplitude of first 3 mmHg and then 10 mmHg. Note how the reference pressure is kept at the same amplitude for all frequencies while the P_M_ is increasingly damped for higher frequencies. Also note the noisy signal from the P_M_ during the measurements of constant pressure.

In the second protocol (Fig. 7), we investigated how catheter length affects the transfer function of a sinusoidal pressure signal. Two combinations of mean pressure and amplitudes were investigated: Mean pressure was set at ~11 mmHg^8^ and amplitudes of 3 mmHg and 10 mmHg were investigated. To test for frequency dependence, both amplitude measurements were repeated with the sinusoidal pressure signal having frequency of 0.5, 1.0, 1.5, 2.0, 3.0, 4.0 and 5.0 Hz. Each frequency was maintained for 20 s. To perform a quality check, each series was initiated and ended with a test of static mean pressure by pressurizing with a hydrostatic column varying in sequence from 0 to −5, 10, and 20 mmHg (Fig. 7). The same sensor was used in all experiments while 5 new lumbar catheters were tested and their frequency dependency was evaluated for 5 different lengths of the catheter, from full length of 84 cm down to 44 cm. To prevent air from entering the system when reducing the catheter length, the disconnection and reconnection of the tubing before and after cutting the catheter were performed under water. In addition to the protocol, in the results we present measurements from one series of similar measurements with a 5 mmHg amplitude where we identified an air bubble in the sensor.

Finally, in a third protocol we did a 3-month stability test with six M.Scio sensors at room temperature and atmospheric air pressure with 18 assessments on each sensor over 40 days and an additional series of 6 measurements between day 80 and 90. Variability with respect to atmospheric pressure (recorded both from the TRU and from the local airport weather station) and ambient temperature (measured with a DeltaOhm HD 9214, Padova, Italy) were evaluated.

P_M_ was recorded to a memory card and then imported into a computer using ICPicture (Miethke GmbH, Potsdam, Germany). From ICPicture it was exported in CSV format. Subsequent signal analysis was performed in MatLab. Both P_M_ and P_Ref_ was digitally filtered with a 5^TH^ order lowpass filter with a cutoff frequency of 10 Hz. We calculated the mean pressures recorded under static pressure conditions of 0, −5, 10, and 20 mmHg. In the time-series analysis an automatic algorithm identified max and min points in the pulse waves and calculated amplitudes and mean pressure. Ten seconds of each 20-second interval (approximately between 9 and 19 s into the interval) were used for detecting peak to peak amplitudes (AMP), mean amplitudes and their standard deviations were estimated for both AMP_M_ and AMP_Ref_ amplitudes. Frequency response was always analyzed as a ratio between AMP_M_ and AMP_Ref_ amplitudes, producing a transfer function.

## Data Availability

The data is available on: https://github.com/anderseklundumu/ICP-monitoring-in-space.
